# Inactivation of α_2_-Macroglobulin by Activated Human Polymorphonuclear Leukocytes

**DOI:** 10.1155/S0962935194000141

**Published:** 1994

**Authors:** G. Deby-Dupont, J.-L. Croisier, G. Camus, D. Brumioul, M. Mathy-Hartert, D. Sondag, C. Deby, M. Lamy

**Affiliations:** 1 Department of Anesthesiology Centre Hospitalier Universitaire, B35 Belgium; 2 Center for the Biochemistry of Oxygen Institut de Chimie, B6 Belgium; 3 lnstitut Supérieur d'Education Physique et de Kinésithérapie B21 Belgium; 4 lnstitut Provincial d'Enseignement Supérieur Paramédical Liège Belgium; 5 Centre de Transfusion Sanguine Centre Hospitalier Universitaire B35, Université de Liège Sart Tilman Liège 4000 Belgium

## Abstract

The proteolytic activity of trypsin releases the dye Remazol Brilliant Blue from its high molecular weight substrate, the skin powder (Hide Powder Azure, Sigma), with an increase in absorbance at 595 nm. Active α_2_- macroglobulin (80 μg/ml) totally inhibits the proteolytic activity of trypsin (14 μg/ml) by trapping this protease. But after a 20 min incubation of α_2_-macroglobulin at 37°C with 2 × 10^6^ human polymorphonuclear leukocytes activated by N-formyl-L-methionyl-L-leucyl-L-phenylalanine (10^−7^ M) and cytochalasin B (10^−8^ M), 100% of trypsin activity was recovered, indicating a total inactivation of α_2_-macroglobuHn. Incubation with granulocyte myeloperoxidase also inactivates α_2_-macroglobulin. Hypochlorous acid, a by-product of myeloperoxidase activity, at a concentration of 10^−7^ M also inactivates α_2_-macroglobulin, which indicates that an important cause of α_2_-macroglobulin inactivation by activated polymorphonuclear leukocytes could be the activity of myeloperoxidase.


Mediators of Inflammation 3, 117–123 (1994)

### Inactivation of $\alpha_2$-macroglobulin by activated human polymorphonuclear leukocytes

G. Deby-Dupont,[1,2,CA] J.-L. Croisier,[3] G. Camus,[2,3] D. Brumioul,[4] M. Mathy-Hartert,[2] D. Sondag,[5] C. Deby[2] and M. Lamy[1,2]

[1]Department of Anesthesiology, Centre Hospitalier Universitaire, B35, [2]Center for the Biochemistry of Oxygen, Institut de Chimie, B6, [3]Institut Supérieur d'Education Physique et de Kinésithérapie, B21, [4]Institut Provincial d'Enseignement Supérieur Paramédical, Liège and [5]Centre de Transfusion Sanguine, Centre Hospitalier Universitaire, B35, Université de Liège, Sart Tilman, 4000 Liège, Belgium

[CA] Corresponding Author

**THE proteolytic activity of trypsin releases the dye Remazol Brilliant Blue from its high molecular weight substrate, the skin powder (Hide Powder Azure, Sigma), with an increase in absorbance at 595 nm. Active $\alpha_2$-macroglobulin (80 μg/ml) totally inhibits the proteolytic activity of trypsin (14 μg/ml) by trapping this protease. But after a 20 min incubation of $\alpha_2$-macroglobulin at 37°C with $2 \times 10^6$ human polymorphonuclear leukocytes activated by N-formyl-L-methionyl-L-leucyl-L-phenylalanine ($10^{-7}$ M) and cytochalasin B ($10^{-8}$ M), 100% of trypsin activity was recovered, indicating a total inactivation of $\alpha_2$-macroglobulin. Incubation with granulocyte myeloperoxidase also inactivates $\alpha_2$-macroglobulin. Hypochlorous acid, a by-product of myeloperoxidase activity, at a concentration of $10^{-7}$ M also inactivates $\alpha_2$-macroglobulin, which indicates that an important cause of $\alpha_2$-macroglobulin inactivation by activated polymorphonuclear leukocytes could be the activity of myeloperoxidase.**

**Key words:** $\alpha_2$-Macroglobulin, Hypochlorous acid, Myeloperoxidase, Neutrophil, Oxygen species, Trypsin

### Introduction

During activation, the polymorphonuclear leukocytes (PMNL) release numerous potent substances, such as active oxygen species, mediators of inflammation and enzymes.[1,2] One of these enzymes, myeloperoxidase (MPO), is included in the primary granules of the polymorphonuclear neutrophils and produces hypochlorous acid from hydrogen peroxide and chloride anion. This oxidant molecule is able to generate chloramines and to destroy bacterial structures resistant to proteinases.[3,4] These potent substances, released by the activated PMNL, normally benefit the host by destroying foreign organisms, and the role of the proteinases would be to allow PMNL to traverse connective tissue barriers by local destruction strictly controlled by antiproteinases.[5]

Matheson *et al.*[6] have discovered that $\alpha_1$-proteinase inhibitor ($\alpha_1$-PI), one of the main plasmatic antiproteinases particularly active against elastase, can be inactivated by MPO in the presence of hydrogen peroxide and chloride anion and that this inactivation results from oxidation into sulphoxide of the two methionyl residues of the reactive site of $\alpha_1$-PI. So, during excessive activation, the neutrophils will release proteinases and MPO together in high concentrations; the oxidative inactivation of $\alpha_1$-PI by MPO-generated factors will result in increasing proteolytic destruction of tissues by proteinases, particularly by free elastase.[7]

$\alpha_2$-Macroglobulin ($\alpha_2$-M) is another important plasma antiproteinase presenting a broad reactivity with serine-, cysteine-, aspartic- and metalloproteinases. Its structure and antiproteinase activity have been studied extensively in the past few years. It possesses four identical subunits linked in pairs by disulphide bonds forming two half molecules which are associated by non-covalent bonds. $\alpha_2$-M contains a bait area of about 35 different amino acids, each being specifically attacked by a particular proteinase.[8–11] By proteolysis of a specific peptide bond of the bait region and cleavage of a thioester bond, the binding of a proteinase leads to a change of $\alpha_2$-M conformation, so that the complex proteinase–$\alpha_2$-M will be recognized quickly by macrophage receptors and rapidly eliminated by the reticulo-endothelial system.[12] This conformational change of $\alpha_2$-M leads to the trapping of the enzyme, which then remains active only against low molecular weight substrates, since by steric inhibition the access to the trapped proteinase is prohibited for high molecular weight substrates.[8,13] It has been demonstrated that $\alpha_2$-M is vulnerable to ammonium salts, which cause the transition of $\alpha_2$-M from the S-form to the F-form that differ in electrophoretic mobility ('slow' for S, and 'fast' for F) and that this transition renders $\alpha_2$-M unable to protect large molecular weight substrates from proteolysis.[14,15] To test the antiproteinase activity of $\alpha_2$-M, Barrett *et al.*[14] assayed the proteolytic activity of

© 1994 Rapid Communications of Oxford Ltd trypsin against a large molecular weight substrate in the presence of $\alpha_2$-M, so that the trapping of trypsin by $\alpha_2$-M reduced or totally inhibited the enzymatic activity. But the pre-treatment of $\alpha_2$-M by ammonium salts (such as methyl ammonium chloride) impaired the inhibitory power of $\alpha_2$-M on trypsin.

The purpose of this study was to demonstrate that $\alpha_2$-M is highly sensitive to oxidation and that activated PMNL or some of their products of activation can destroy its antiproteinase activity, as they do for $\alpha_1$-PI.[6,7] The results are in agreement with those reported previously by Reddy *et al.*[16] Taken together, these findings confirm Weiss' hypothesis of a possible oxidation of $\alpha_2$-M by activated neutrophils with possible dramatic consequences on tissue destruction during acute inflammation.[5]

### Materials and Methods

*Materials:* The chemicals reagents (analytical grade) were purchased as follows: trihydroxyl methyl amino methane (Tris), sodium and potassium chloride, calcium and magnesium chloride, sodium dihydrogeno- and potassium monohydrogenophosphate, ammonium sulphate, hydrogen peroxide ($H_2O_2$), hypochlorous acid (HOCl), dimethylsulphoxide, glucose, toluene and Brij 35 from Merck Chemical; methylammonium chloride from Janssen Chemica; N-formyl L-methionyl L-leucyl-L-phenylalanine (FMLP), cytochalasin B, tetrazolium nitroblue, superoxide dismutase, cetyltrimethylammonium bromide, thiourea and hide powder azure from Sigma; bovine trypsin, soybean trypsin inhibitor (SBTI), bovine $\alpha_2$-macroglobulin and Chromozym TRY (carbobenzoxy-L-valyl-L-glycyl-L-arginyl *p*-nitroanilide) from Boehringer; Dextran T500, Ficoll–Paque; Sephadex SP50 from Pharmacia Fine Chemical; and Aca 34 from IBF.

The bovine trypsin preparation was standardized by active site titration[17] and was found to contain 83% active enzyme. The trypsin concentrations in the assays are expressed as active trypsin. The purity of trypsin, $\alpha_2$-macroglobulin and myeloperoxidase were controlled by SDS–polyacrylamide gel electrophoresis (3–15%).

*Preparation and activation of polymorphonuclear leukocytes:* Polymorphonuclear leukocytes (PMNL) were isolated from human blood of healthy donors following the technique of Borgeat and Samuelsson.[18] After a first centrifugation (25 min at 200 × ***g***), the plasma was discarded and the 'buffy coat' was resuspended in one volume of saline and a half volume of 6% Dextran T 500 in saline, until erythrocytes settled down. The 'foamy coat' was layered over Ficoll–Paque. The PMNL pellets were collected after centrifugation (100 × ***g***, 20 min at 20°C) and briefly exposed to Tris (2.06%)–ammonium chloride (0.83%) at pH 7.4, to lyse any remaining erythrocytes. After a last centrifugation (15 min at 20°C, 200 × ***g***), the cells were adjusted to $20 \times 10^6$ cells/ml of phosphate buffered saline (PBS: $Na_2HPO_4$, $10^{-2}$ M; $KH_2PO_4$, $1.5 \times 10^{-3}$ M; KCl, $2.7 \times 10^{-3}$ M; NaCl, 0.14 M; glucose, $7.5 \times 10^{-3}$ M, pH 7.3) for further use.

For activation, $2 \times 10^6$ cells (in 0.1 ml PBS) were treated with 10 μl of FMLP (100 μg/ml), 10 μl of cytochalasin B (250 μg/ml) and 10 μl of a 200 mM $CaCl_2$–50 mM $MgCl_2$ solution in PBS. In order to prove the PMNL activation by FMLP and cytochalasin B, we used the superoxide dismutase inhibitable reduction of either tetrazolium nitroblue (NBT) or ferricytochrome C to measure the rate of superoxide anion generation by activated cells.[1,19]

$2 \times 10^6$ PMNL in 0.1 ml PBS and 0.9 ml of either a $10^{-3}$ M NBT or ferricytochrome C solution were incubated for 5 min at 37°C. After addition of FMLP and cytochalasin B, the absorbance at 522 or 550 nm was followed and compared with the same assay in the presence of 60 μg of superoxide dismutase, the specific enzyme destroying the superoxide anion.

Before the test on the activity of trypsin, $\alpha_2$-M was preincubated with PMNL for 20 min at 37°C in a final volume of 1 ml ($\alpha_2$-M final concentration, 800 μg/ml). Control assays were made by incubation of $\alpha_2$-M in the same conditions but with non-activated PMNL. After centrifugation (600 × ***g***, 5 min at 20°C), the supernatants were pipetted and used for trypsin activity measurement. Control experiments of the direct action of activated or non-activated PMNL on trypsin activity were carried out.

To rule out a possible release of a trypsin-like enzyme by activated PMNL, which would interfere with the release of the dye from the substrate, we looked at a possible trypsin-like activity in the supernatant of PMNL by using a low molecular weight chromogenic substrate, Chromozym TRY® (carbobenzoxy-L-valyl-L-glycyl-L-arginyl-*p*-nitroanilide).[20] Trypsin cleaves this substrate releasing *p*-nitroaniline, the absorbance of which is determined at 405 nm. The working solution of Chromozym TRY® was prepared by dilution of 2 ml of a concentrated solution (1.5 mg/ml of dimethylsulphoxide) in 25 ml of Tris–HCl buffer (0.1 M, pH 8.0) added to $CaCl_2$ (0.02 M). In the assay tube, 1 ml of the working solution of Chromozym TRY® is mixed with 0.8 ml of Tris–HCl buffer and 0.2 ml of the supernatant from activated PMNL. After 10 min of reaction, the absorbance was measured against the same test without supernatant (control test). A standard curve was established with concentrations of bovine trypsin ranging from 2 to 100 ng/ml.

*Effects of various substances on $\alpha_2$-macroglobulin activity:* MPO was purified from PMNL isolated from 30 l of blood from healthy donors, according to the method described previously.[21] The purity of the final preparation was checked by the ratio of absorbance at 430 nm *vs.* 280 nm and by analytical electrophoresis either on 9% polyacrylamide gel at pH 4.6 or on gradient polyacrylamide gel (7–15%) in the presence of 0.1% sodium dodecyl sulphate and 2-mercapto-ethanol, in 0.1 M Tris–glycine buffer at pH 8.3.[22]

To demonstrate a possible role played by MPO in $\alpha_2$-M inactivation, the following experiments were carried out. Forty µl of MPO ($1.25 \times 10^{-6}$ M in phosphate buffer 0.1 M, NaCl 0.2 M), 10 µl of $H_2O_2$ ($10^{-4}$ M) and 50 µl of $\alpha_2$-M (1600 µg/ml in Tris–HCl buffer) were incubated for 10 min at 37°C. 100 µl of trypsin (14 µg/ml in Tris–HCl buffer) were then added, the volume of the assay tube was adjusted to 0.5 ml with Tris–HCl buffer and a new incubation was performed for 10 min at 37°C. Next, the proteolytic activity of trypsin was assayed as described above. To test that the trypsin activity was not directly inhibited by MPO or $H_2O_2$, the same assays were performed in the absence of $\alpha_2$-M (control assay).

The enzymatic activity of MPO produces oxidant chlorinated species, particularly hypochlorous acid (HOCl) which could play a role in $\alpha_2$-M inactivation. So, its direct action on $\alpha_2$-M was tested. In each assay, 50 µl of $\alpha_2$-M (1600 µg/ml) and 50 µl of HOCl ($10^{-4}$M) were incubated for 5 min at 37°C. One hundred µl of trypsin (14 µg/ml) were added to the assay tube and the volume adjusted to 0.5 ml with Tris–HCl buffer. After 10 min incubation at 37°C, the proteolytic activity of trypsin was tested. Control assays were made without $\alpha_2$-M.

The effects of thiourea, an inhibitor of MPO activity, was assessed by incubating PMNL ($20 \times 10^6$ cells/ml) for 5 min at 37°C with 10 µl of a thiourea solution ($10^{-4}$ M).[23] The cells were then activated in the presence of $\alpha_2$-M (see above). After incubation for 20 min at 37°C, the supernatants were used to test the remaining inhibiting activity of $\alpha_2$-M on trypsin activity. Control tests were made in the same manner with all the reagents except $\alpha_2$-M.

*Assay of proteinase inhibitory activity of $\alpha_2$-macroglobulin:* The activity of $\alpha_2$-M was determined as its inhibition on the proteolytic activity of trypsin against a large molecular weight substrate, hide powder azure.[14] Free trypsin breaks the link between the hide powder and a dye, Remazol Brilliant Blue, releasing it in the supernatant where its absorbance is determined at 595 nm ($A_{595}$). When complexed by $\alpha_2$-M, trypsin is inactive against this substrate.

All the assays were prepared in triplicate and the reagents were dissolved in the test buffer, 0.1 M Tris–HCl at pH 8.1 added to 0.02 M $CaCl_2$ and 0.1% Brij 35. In each of the triplicate assay tubes, 0.1 ml of trypsin (14 µg/ml) was incubated for 10 min at 37°C with 0.1 ml of $\alpha_2$-M (800 µg/ml) in a total volume of 0.5 ml made up with the test buffer. Water (0.5 ml) was then added to the tube, followed by 0.8 ml of the substrate suspension (12.5 mg of hide powder azure per ml in 0.6 M sucrose, 0.1% Brij 35, 0.03% toluene). After an incubation of 20 min at 37°C with continuous shaking, 1 ml of water was added and after centrifugation (2 000 × **g** for 5 min), the $A_{595}$ of supernatant was determined.

To verify the existence of a linear relationship between increasing concentrations of trypsin and the amount of the dye released from a constant concentration of the substrate, a standard curve of 4 to 20 µg of trypsin/ml was determined in the absence of $\alpha_2$-M. In the same manner, to verify the existence of a linear relationship between increasing amounts of $\alpha_2$-M and the inhibition of trypsin activity, a constant concentration of trypsin was incubated with increasing amounts of $\alpha_2$-M from 100 to 1 000 µg/ml.

Soybean trypsin inhibitor (SBTI) is an efficacious inhibitor of trypsin that blocks the active centre of the enzyme.[24] Consequently, when preincubated with trypsin (5 min at 20°C), it has to prevent the enzyme action on the hide powder azure substrate. SBTI was used in these experiments to prove the validity of the test assay of trypsin enzymatic activity.

Methylammonium chloride causes the cleavage of the thioester bonds which link the four sub-units of $\alpha_2$-M. This cleavage changes the $\alpha_2$-M conformation, leading to $\alpha_2$-M inactivation.[14,15] Methylammonium chloride (in concentrations between 20 and 400 mM) was preincubated with $\alpha_2$-M (800 µg/ml) for 30 min at 20°C. Trypsin activity was then tested in the presence of this inactivated $\alpha_2$-M, following the previously described technique. Control assays were carried out in the absence of $\alpha_2$-M to measure the direct influence of methylammonium chloride on trypsin activity.

### Results

Using the technique of Barrett,[14] a linear relationship was found between the amount of trypsin incubated in the presence of a fixed concentration of the substrate (10 mg) and the amount of the released dye, for trypsin concentrations ranging from 2 to 20 µg/ml ($A_{595}$ from 0.05 to 0.32). For further assays, a fixed trypsin concentration of 14 µg/ml was chosen giving a reproducible $A_{595}$ of 0.22 ± 0.02 ($n = 30$). The $A_{595}$ obtained for this trypsin concentration in the absence of $\alpha_2$-M was taken as 100% of trypsin activity (Fig. 1, Column 1). The enzyme assay was affected by the trypsin inhibitor SBTI, and the trypsin activity (14 µg/ml) was completely inhibited for an SBTI concentration of 15 µg/ml, which represents an SBTI/trypsin molar ratio of 1.1 (Fig. 1, Column 6). A linear relationship was observed between increasing

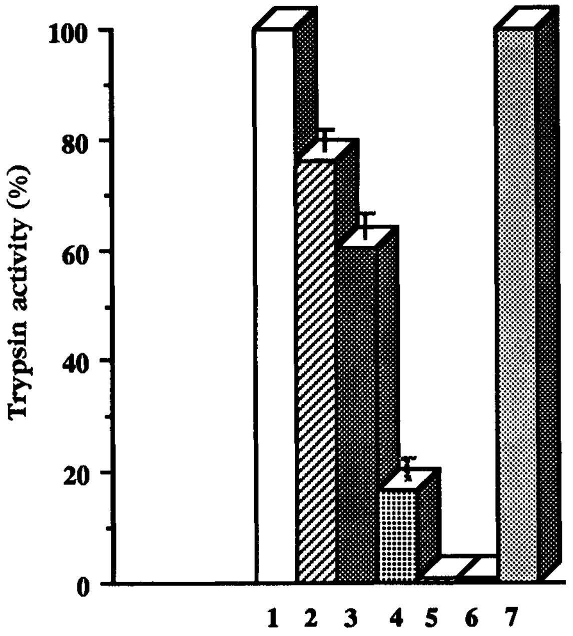

FIG. 1. Role of $\alpha_2$-macroglobulin ($\alpha_2$-M) and soybean trypsin inhibitor (SBTI) on trypsin activity. Column 1, trypsin alone (14 $\mu$g/ml); Columns 2, 3, 4 and 5, increased concentrations of $\alpha_2$-M (200, 400, 600, 800 $\mu$g/ml); Column 6, soybean trypsin inhibitor (15 $\mu$g/ml); Column 7, $\alpha_2$-M (800 µg/ml) inactivated by methylammonium chloride (400 mM).

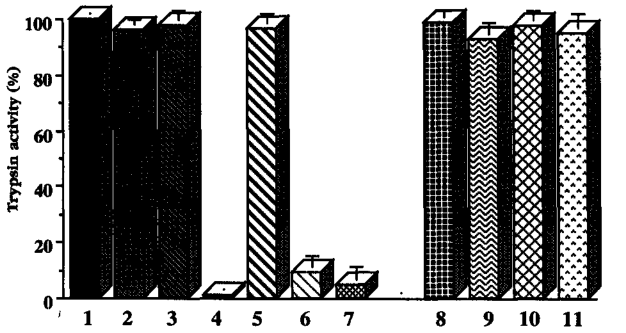

FIG. 3. Role of PMNL and MPO in $\alpha_2$-M inactivation. Column 1, trypsin alone (14 $\mu$g/ml); Column 2, trypsin + supernatant of activated PMNL; Column 3, trypsin + supernatant of non-activated PMNL; Column 4, trypsin + $\alpha_2$-M (800 $\mu$g/ml); Column 5, trypsin + $\alpha_2$-M treated by supernatant of activated PMNL; Column 6, trypsin + $\alpha_2$-M treated by supernatant of non-activated PMNL; Column 7, trypsin + $\alpha_2$-M treated by activated PMNL preincubated with thiourea ($10^{-4}$M); Column 8, trypsin + MPO ($10^{-7}$M); Column 9, trypsin + HOCl ($10^{-7}$M); Column 10, trypsin + $\alpha_2$-M treated by MPO; Column 11, trypsin + $\alpha_2$-M treated by HOCl ($10^{-7}$M).

concentrations of $\alpha_2$-M (from 200 to 800 $\mu$g/ml) and the activity of trypsin (fixed concentration of trypsin, 14 $\mu$g/ml) (Fig. 1, Columns 2, 3, 4 and 5). The enzyme activity was completely abolished for an $\alpha_2$-M concentration of 800 µg/ml, which represents a trypsin/$\alpha_2$-M ratio of 2 on the basis of the trypsin and $\alpha_2$-M molecular weights of 23 and 725 kDa, respectively. This concentration (800 µg/ml $\alpha_2$-M) was chosen for further assay. When preincubated with $\alpha_2$-M, methylammonium chloride blocks the antiproteinase activity of $\alpha_2$-M. A complete inactivation of $\alpha_2$-M (800 µg/ml) was observed for a methylammonium chloride concentration of 400 mM (Fig. 1, Column 7). At the same concentration, methylammonium chloride was totally inactive on trypsin alone.

Fig. 2 shows the increasing production of superoxide anion by PMNL activated by FMLP and cytochalasin B. The absorbance of formazan blue (produced by reaction of tetrazolium nitroblue with

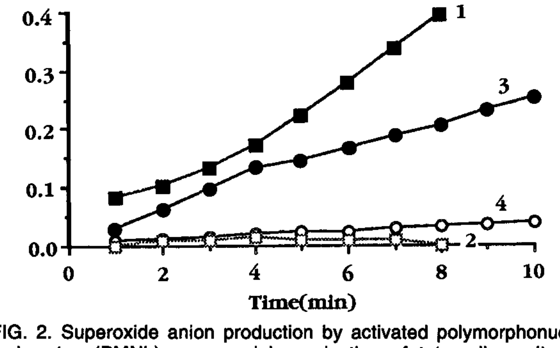

FIG. 2. Superoxide anion production by activated polymorphonuclear leukocytes (PMNL) measured by reduction of tetrazolium nitroblue (Curves 1 and 2, at 592 nm) or ferricytochrome C (Curves 3 and 4, at 550 nm). ● or ■, activated PMNL (activators: FMLP 1.0 µg and cytochalasin B 2.5 µg). O or □, non-activated PMNL.

the superoxide anion generated by PMNL) increased with time for activated PMNL, remaining very low for non-activated cells. Fig. 2 also shows the progressive reduction of ferricytochrome C by anion superoxide produced by activated PMNL after a short period of latency. The production of superoxide anion by non-activated cells is given as comparison. For some PMNL preparations, superoxide anion was produced (at low levels) without further addition of FMLP and cytochalasin B, indicating a possible unexpected activation of the cells during their isolation from blood. PMNL preparations with this unexpected activation were not used for our experiments of $\alpha_2$-M inactivation.

Using the chromozym TRY® technique, proteolytic activity sufficient for interfering with the assay conditions could not be detected. Additionally, by themselves, supernatants of activated or non-activated PMNL were unable to release the dye from the substrate. Moreover they did not decrease the release of the dye from the substrate, which indicates that no trypsin inhibitor was produced (Fig. 3, Columns 2 and 3). Activated PMNL destroyed the antiproteinase activity of $\alpha_2$-M (Fig. 3, Column 5) since trypsin activity was completely recovered after treatment of $\alpha_2$-M by activated PMNL. This $\alpha_2$-M inactivation was dependent on the activation of PMNL, for non-activated PMNL did not affect the inhibitory power of $\alpha_2$-M on trypsin (Fig. 3, Column 6). When thiourea was added (at the concentration of $10^{-4}$ M) to the incubation medium of $\alpha_2$-M with activated PMNL, it prevented the inactivation of $\alpha_2$-M, resulting in a complete recovery of $\alpha_2$-M inhibition on trypsin activity (Fig. 3, Column 7).

The treatment of $\alpha_2$-M by MPO in the presence of $H_2O_2$ and $Cl^-$ inactivated $\alpha_2$-M since all the activity of trypsin was recovered (Fig. 3, Column 10), while the enzyme was not directly affected by the activity of MPO (Fig. 3, Column 8). Hypochlorous acid, HOCl,

**Table 1.** Role of $H_2O_2$ and HOCl on trypsin activity

| Concentration of oxidant (M) | Trypsin activity (%) |
|---|---|
| $H_2O_2$ | |
| 0 | 100 |
| $10^{-7}$ | 100 |
| $10^{-6}$ | 100 |
| $10^{-5}$ | 100 |
| HOCl | |
| 0 | 100 |
| $10^{-7}$ | 90 |
| $10^{-6}$ | 72 |
| $10^{-5}$ | 0 |
| $10^{-4}$ | 0 |

the direct product of MPO activity, was tested alone. At a concentration of $10^{-7}$ M, it destroyed the activity of $\alpha_2$-M (Fig. 3, Column 11). However, as HOCl is very oxidant, it was tested directly on trypsin activity. At concentrations of $10^{-5}$ and $10^{-4}$ M, it destroyed the enzyme (Table 1). But in our assay conditions HOCl ($10^{-7}$ M), had little effect on trypsin activity (Fig. 3, Column 9). Hydrogen peroxide, at concentrations between $10^{-7}$ and $10^{-4}$ M, did not inactivate trypsin (Table 1).

When $\alpha_2$-M is inactivated, its antigenic activity shows little change. Using the immunoprecipitin analysis technique (immunoelectropherogram), a difference in mobility of the inactive human $\alpha_2$-M, compared with its native active form (Fig. 4), was observed. A similar effect was observed by Hubbard *et al.* for complexed $\alpha_2$-M[25] and by Barrett *et al.*[14] who, using immunoprecipitin analysis, also found a slight difference. The heights of rockets for the F-$\alpha_2$-M (fast form or complexed by proteinases) were lower (12 to 29%) than those for the S-$\alpha_2$-M (uncomplexed form), depending on the antiserum.

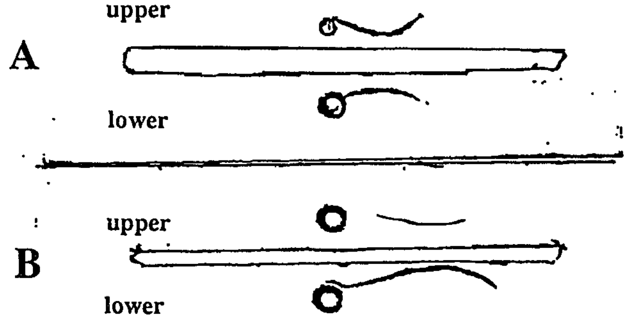

FIG. 4. Immunoprecipitin analysis (immunoelectropherogram) of human $\alpha_2$-M (anode at right). Troughs, rabbit antiserum obtained against human $\alpha_2$-M. Panel A, upper well, normal human plasma; lower well, pure native $\alpha_2$-M. Panel B, upper well, $\alpha_2$-M treated by activated PMNL; lower well, $\alpha_2$-M treated by human myeloperoxidase. Note the difference in mobility between the slow native $\alpha_2$-M and the fast inactive form after PMNL or myeloperoxidase treatment.

### Discussion

$\alpha_2$-Macroglobulin (about $1.1 \times 10^9$ M) was inactivated by $2 \times 10^6$ PMNL. This inactivation was complete, with a total loss of antiproteinase activity, but needed an activation of the cells, since $\alpha_2$-M treated by non-activated PMNL conserved its antiproteinase activity. The control assays allowed us to exclude the capture of $\alpha_2$-M by the cells or the release of an inhibitor directly acting on trypsin. The activation of PMNL can release proteinases (such as elastase) which, by binding to $\alpha_2$-M, could have rendered it inactive towards trypsin. But it seems difficult to admit that $2 \times 10^6$ cells would have released sufficient amounts of proteinases to completely complex the amount of $\alpha_2$-M used in these assays.

When PMNL are activated, they undergo the 'respiratory burst', triggering the activity of membrane NADPH oxidase leading to the production of excited forms of oxygen, particularly $O_2$ (superoxide anion) from which $H_2O_2$ can be generated.[1,26] PMNL activation also leads to degranulation with the release of proteolytic and hydrolytic enzymes from granules. MPO is an important enzyme present in the azurophilic granules. It uses $H_2O_2$ and halide anions ($Cl^-$, $I^-$ etc.) for its enzymatic activity, which generates various strong oxidants, such as HOCl and chloramines.[27,28] The inactivation of $\alpha_2$-M by excited PMNL can be due to the excited forms of oxygen or the action of the granulocytic enzymes. It is unlikely that $O_2$ is responsible for $\alpha_2$-M inactivation. To attack $\alpha_2$-M, $O_2$ needs to reach this molecule, but, in our assay conditions, its access to $\alpha_2$-M is limited by its instability in aqueous media. Moreover, the effects of $O_2$ on $\alpha_2$-M were tested using the production of $O_2$ by an acetaldehyde–xanthine oxidase system[29] in buffer with added $\alpha_2$-M. In these conditions, the antiproteinase activity of $\alpha_2$-M remained unaffected. Consistent with the conclusion that $O_2$ was not the primary species involved are the results of a previous study by Reddy *et al.*[16] where neutrophil cytoplasts, able to generate $O_2$ and $H_2O_2$ but not HOCl, failed to inactivate $\alpha_2$-M in the absence of exogenous MPO. By dismutation, $O_2$ produces $H_2O_2$ which could directly act on $\alpha_2$-M. However, the use of $H_2O_2$ concentrations between $10^{-7}$ and $10^{-5}$ M failed to inactivate $\alpha_2$-M.

Since neither $O_2$ nor $H_2O_2$ appears responsible for the destruction of $\alpha_2$-M antiproteinase activity, attention was therefore focused on the role of MPO. The activation of PMNL leads to their degranulation, with activation of many enzymes. In presence of $H_2O_2$ produced from $O_2$ dismutation, MPO generates potent oxidant compounds, particularly HOCl. It has been shown that the activity of MPO inactivates $\alpha_1$-PI.[6,7] This study demonstrates that $\alpha_2$-M is inactivated in a similar manner by MPO and that the main product of MPO activity, HOCl, is responsible for this inactivation. The results are in agreement with those obtained by Reddy *et al.*[16] These authors showed that human $\alpha_2$-M incubated in the presence of activated PMNL rapidly lost the ability to inhibit neutrophil and pancreatic elastase activity. They also showed that PMNL inactivate $\alpha_2$-M by a HOCl dependent process. However, it appears from our observations that this inactivation of $\alpha_2$-M by HOCl is nonspecific, since trypsin itself can be totally destroyed by HOCl, but only at higher concentrations ($10^{-5}$M) of this molecule. In the same manner, $H_2O_2$ also has a destroying effect on $\alpha_2$-M, but hydrogen peroxide at concentrations of $10^{-2}$M is needed for this destruction. It is probable that inactivation of $\alpha_2$-M occurs by the destruction of the S–S bonds between the four subunits of the protein, leading to their dissociation. The limited reduction of $\alpha_2$-M produces monomers (180 kDa) which reassociate after proteinase treatment but do not prevent proteinases from cleaving hide powder azure.[14] Larsson *et al.*[30] found that enzymatic treatment of $\alpha_2$-M cleaves the disulphide bonds inhibiting the further binding of trypsin. Moncino *et al.*,[31] by chemical reduction of $\alpha_2$-M with dithiotreitrol, obtained $\alpha_2$-M monomers retaining their proteinase binding capacity, but becoming unable to inhibit the enzymatic activity of the bound proteinases. By electrophoresis, we confirmed that, after treatment by activated PMNL or MPO, the electrophoretic pattern of $\alpha_2$-M was modified with the disappearance of the 720 000 kDa band and the presence of new bands localized around 180 000 kDa. These assays were carried out using bovine $\alpha_2$-M, as well as $\alpha_2$-M purified from human plasma. This human $\alpha_2$-M, when treated by activated PMNL or MPO, loses its trypsin inhibitory capacity in the same manner as bovine $\alpha_2$-M.

In our experimental conditions, $\alpha_2$-M ($10^{-9}$M) was completely inactivated by a 20 min incubation in the presence of excited PMNL, but $2 \times 10^6$ cells per assay were used. Moreover, these cells remained in close contact with $\alpha_2$-M for a long time. While PMNL can exert strong destructive effects on host tissues,[5,32,33] our experimental conditions are far from being comparable to a normal *in vivo* situation, where plasma protectors will counteract any PMNL destroying activity on $\alpha_2$-M. Therefore, the extent to which *in vivo* $\alpha_2$-M inactivation by triggered PMNL could occur and contribute to tissue destruction through imbalance between proteases and antiproteases is still obscure and requires further studies. It is worth remembering, however, that we have measured important plasma concentrations of MPO in inflammation states associated with ARDS, sepsis or acute pancreatitis.[34] On the other hand, decreased plasma and serum concentrations of $\alpha_2$-M have been reported in ARDS and septic patients. Furthermore, these decreased plasma or serum concentrations of $\alpha_2$-M are measured by routine immunological or immunoelectrophoresis techniques that are unable to easily differentiate the active from the inactive form of $\alpha_2$-M, so that the plasma or serum concentrations of the protein measured by these techniques do not reflect the capacity of $\alpha_2$-M to inhibit proteases. Taken together, these findings indicate that *in vivo* inactivation of $\alpha_2$-M cannot be ruled out, but as suggested above the occurrence of this phenomenon and its possible involvement in tissue destruction by active protease in inflammatory diseases remain to be firmly demonstrated.

### References

1. Babior BM, Kipnes RS, Curnutte JT. Biological defense mechanisms: the production by leukocytes of superoxide, a potential bactericidal agent. *J Clin Invest* 1973 **52**: 741–744.
2. Henson PM, Henson JE, Fittschen C, Kimani G, Bratton DL, Riches DWH. Phagocytic cells: degranulation and secretion. In: Gallin I, Goldstein IM, Snyderman R, eds. *Inflammation: Basic Principles and Clinical Correlates*, New York: Raven Press, 1988; 362–390.
3. Klebanoff SJ. Myeloperoxidase–halide–hydrogen peroxide antibacterial system. *J Bacteriol* 1968; **95**: 2131–2138.
4. Weiss SJ, Lampert MB, Test ST. Long-lived oxidants generated by human neutrophils: characterization and bioactivity. *Science* 1983; **222**: 625–628.
5. Weiss SJ. Tissue destruction by neutrophils. *New Engl J Med* 1989; **320**: 365–376.
6. Matheson NR, Wong PS, Travis J. Enzymatic inactivation of human-$\alpha_1$-proteinase inhibitor. *Biochem Biophys Res Comm* 1979; **88**: 402–409.
7. Clark RA, Stone PJ, El Hag AZ, Calore JD, Franzblau C. Myeloperoxidase-catalyzed inactivation of $\alpha_1$-proteinase inhibitor by human neutrophils. *J Biol Chem* 1981; **256**: 3348–3353.
8. Barrett AJ, Starkey PM. The interaction of $\alpha_2$-M with proteinases. *Biochem J* 1973; **133**: 709–724.
9. Roberts RC, Hall PK. Specificity of proteinases for the bait region of $\alpha_2$-M. In: Feinman RD, ed. *Chemistry and Biology of* $\alpha_2$*-M, Vol 421*. New York: Annals of the New York Academy of Sciences, 1983; 61–68.
10. Bretaudière P, Tapon-Bretaudière J, Stoops JK. Structure of native $\alpha_2$M and its transformation to the protease bound form. *Proc Natl Acad Sci USA*. 1988; **85**: 1437–1441.
11. Sottrup-Jensen L, Lonblad PB, Stepanik TM, Petersen TE, Magnusson S, Jornvall H. Primary structure of the 'bait' region for proteinases in $\alpha_2$-macroglobulin. *FEBS Lett* 1981; **127**: 167–173.
12. Debanne MT, Beel R, Dolovich J. Uptake of proteinase-$\alpha_2$M complexes by macrophages. *Biochem Biophys Acta* 1975; **411**: 295–304.
13. Bieth JG, Kandel M, Zreika M. Interaction of protein substrate and inhibitors with $\alpha_2$-M-bound proteinase. In: Feinman RD, ed. *Chemistry and Biology of* $\alpha_2$*-M, Vol 421*. New York: Annals of the New York Academy of Sciences, 1983; 209–217.
14. Barrett AJ, Brown AA, Sayers CA. The electrophoretically slow and fast forms of the $\alpha_2$-macroglobulin molecule. *Biochem J* 1979; **181**: 401–418.
15. Steinbuch M, Pejaudier L, Quentin M, Martin V. Molecular alteration of $\alpha_2$-macroglobulin by aliphatic amines. *Biochem Biophys Acta* 1968; **154**: 228–231.
16. Reddy VY, Pizzo SV, Weiss SJ. Functional inactivation and structural disruption of human $\alpha_2$-macroglobulin by neutrophils and eosinophils. *J Biol Chem* 1989; **264**: 13801–13809.
17. Chase T, Shaw E. p-Nitrophenyl-p'-guanidinbenzoate HCl: a new active site titrant for trypsin. *Biochem Biophys Res Commun* 1967; **29**: 508–514.
18. Borgeat P, Samuelsson B. Arachidonic acid metabolism in polymorphonuclear leucocytes: effects of ionophore A23187. *Proc Natl Acad Sci USA* 1979; **76**: 2148–2152.
19. Badwey JA, Curnutte JT, Karnovsky ML. Cis-polyunsaturated fatty acids induce high levels of superoxide production by human neutrophils. *J Biol Chem* 1981; **256**: 12640–12643.
20. Cullman W, Dick W. A chromogenic assay for evaluation of $\alpha_2$-macroglobulin level in serum and cerebrospinal fluid. *J Clin Chem Clin Biochem* 1981; **19**: 287–290.
21. Bakkenist ARJ, Wever R, Vulsma T, Plat H, Van Gelder F. Isolation procedure and some properties of myeloperoxidase from human leukocytes. *Biochim Biophys Acta* 1978; **524**: 45–54.
22. Deby-Dupont G, Pincemail J, Thirion A, Deby C, Lamy M, Franchimont P. Self-labeling of human polymorphonuclear leucocyte myeloperoxidase with $^{125}$iodine. *Experientia* 1991; **47**: 952–957.
23. Swenson RP, Howard JB. Characterization of alkylamine-sensitive site in $\alpha_2$-macroglobulin. *Proc Natl Acad Sci* 1979; **76**: 4313–4316.
24. Kassell B. Trypsin and chymotrypsin inhibitors from soybeans. In: Lorand L, ed. *Methods in Enzymology, Vol 19*. New York: Academic Press. 1970: 853–862.
25. Hubbard WJ, Hess AD, Hsia S, Amos DB. The effects of electrophoretically 'slow' and 'fast' $\alpha_2$-macroglobulin on mixed lymphocyte cultures. *J Immunol* 1981; **126**: 292–299.
26. Root RK, Metcalf JA. $H_2O_2$ release from human granulocytes during phagocytosis: relationship to superoxide anion formation and cellular catabolism of $H_2O_2$: studies with normal and cytochalasin B treated cells. *J Clin Invest* 1977; **60**: 1266–1279.

27. Bakkenist ARJ, De Boer JEG, Plat H, Wever R. The halide complexes of myeloperoxidase and the mechanism of the halogenation reactions. *Biochim Biophys Acta* 1980; **613**: 337–348.
28. Thomas EL, Grisham MB, Melton DF, Jefferson MM. Evidence for a role of taurine in the *in vitro* oxidative toxicity of neutrophils towards erythrocytes. *J Biol Chem* 1985; **260**: 3321–3329.
29. Kellogg EW, Fridovich I. Superoxide, hydrogen peroxide and singlet oxygen in lipid peroxidation by a xanthine oxidase system. *J Biol Chem* 1975; **250**: 8812–8817.
30. Larsson LJ, Holmgren A, Medstrød B, Lindblom T, Björk I. Subunits of $\alpha_2$-macroglobulin produced by specific reduction of interchain disulfide bonds with thioredoxin. *Biochemistry* 1988; **27**: 983–991.
31. Moncino MD, Roche PA, Pizzo SV. Characterization of human $\alpha_2$-macroglobulin monomers obtained by reduction with dithiothreitol. *Biochemistry* 1991; **30**: 1545–1551.
32. Warren JS, Warp PA, Johnson KJ. Mechanisms of damage to pulmonary endothelium. In: Ryan US, ed. *Pulmonary Endothelium in Health and Disease, Vol 32.* New York: Marcel Dekker, 1987; 107–120.
33. Vercellotti M. Role of neutrophils in endothelial injury. In: Bihari DJ, Cerra FB, eds. *Multiple Organ Failure, Vol 5.* Fullerton: The Society of Critical Care, 1990; 77–91.
34. Pincemail J, Deby-Dupont G, Lamy M. Role of neutrophils in critically ill patients: myeloperoxidase, a specific marker of their activation. In: Vincent JL, ed. *Update in Intensive Care and Emergency Medicine, Vol 8.* Berlin: Springer-Verlag, 1989; 24–32.

ACKNOWLEDGEMENTS. The authors would like to thank Mrs M. Dister, Mrs J. Neuray and Mrs Dethier for their excellent technical assistance and Mrs V. Casertano and Mr D. Jeuniaux for their secretarial help. This work was supported by the Fonds National de la Recherche Scientifique (FNRS) grants nos. 3.4569.89 and 3.4574.89.

**Received 21 October 1993; accepted in revised form 20 December 1993**
